# Cardiovascular disease risk models and dementia or cognitive decline: a systematic review

**DOI:** 10.3389/fnagi.2023.1257367

**Published:** 2023-10-12

**Authors:** Ruirui Jia, Qing Wang, Hengyi Huang, Yanli Yang, Yuet Foon Chung, Tao Liang

**Affiliations:** ^1^School of Nursing, Lanzhou University, Lanzhou, China; ^2^School of Nursing, Chinese Academy of Medical Sciences and Peking Union Medical College, Beijing, China

**Keywords:** cardiovascular disease risk models, cardiovascular health, dementia, cognitive decline, health promotion

## Abstract

**Background:**

Health cognitive promotion and protection is a critical topic. With the world’s aging population and rising life expectancy, there will be many people living with highly age-related dementia illnesses. Cardiovascular disease (CVD) and dementia share the same risk factors, such as unhealthy lifestyles and metabolic factors. These recognized risks associated with CVD and dementia frequently co-occur. CVD risk models may have a close association with dementia and cognitive decline. So, this systematic review aimed to determine whether CVD risk models were connected with dementia or cognitive decline and compare the predictive ability of various models.

**Methods:**

PubMed, Web of Science, PsychINFO, Embase, Cochrane Library, CNKI, Sinomed, and WanFang were searched from 1 January 2014 until 16 February 2023. Only CVD risk models were included. We used the Newcastle-Ottawa scale (NOS) for the quality assessment of included cohort studies and the Agency for Healthcare Research and Quality (AHRQ) for cross-sectional studies. The Preferred Reporting Items for Systematic Reviews and Meta-analysis (PRISMA) statement’s guidelines were followed in this systematic study.

**Results:**

In all, 9,718 references were screened, of which 22 articles were included. A total of 15 CVD risk models were summarized. Except for the Cardiovascular Health in Ambulatory Care Research Team (CANHEART) health index, the other 14 CVD risk models were associated with dementia and cognitive decline. In comparison, different CVD risk models and domain-specific cognitive function correlation variation depended on cohort characteristics, risk models, cognitive function tests, and study designs. Moreover, it needed to be clarified when comparing the predicting performance of different CVD risk models.

**Conclusion:**

It is significant for public health to improve disease risk prediction and prevention and mitigate the potential adverse effects of the heart on the brain. More cohort studies are warranted to prove the correlation between CVD risk models and cognitive function. Moreover, further studies are encouraged to compare the efficacy of CVD risk models in predicting cognitive disorders.

## 1. Introduction

With the aging population and increasing life expectancy, people now have significantly higher chances of developing highly age-related dementia disorders (Livingston et al., 2020). Age-related dementia is caused by a complex combination of hereditary and environmental variables (Fayosse et al., 2020). In low- and middle-income nations, the number of people with dementia is increasing faster than in high-income countries, owing to a greater risk factor burden (Livingston et al., 2020). With less knowledge of the underlying pathology and no targeted treatments, modifiable factors have drawn particular attention for dementia prevention. According to the World Health Organization, health cognitive promotion and protection is a public health priority to achieve the goal of universal cognitive health coverage (WHO, 2020).

Increasing evidence demonstrates that heart and brain health are inextricably linked (Zhao et al., 2023). Patients diagnosed with cardiovascular disease (CVD) usually had poorer cognition than those without CVD (Covello et al., 2021). According to the most recent descriptions of novel pathological reasons, mixed dementia (Alzheimer’s disease and cerebrovascular injuries) is more common among the oldest seniors (those over 90 years old; Livingston et al., 2020). More and more population-based cohort studies have discovered that dementia and CVD may share the same risk factors. Modifiable health and lifestyle factors such as blood pressure (BP), total cholesterol (TC), blood glucose, physical activity, dietary habits, and smoking could improve vascular health and potentially reduce the risk of cognitive decline and dementia (Rasmussen et al., 2020). Cardiovascular risk factors may hasten cognitive decline by generating cerebral hypoperfusion, hypoxia, embolism, or infarcts, which result in vascular and degenerative brain lesions (Bancks et al., 2019; Lane et al., 2020). According to neuropathological research, dementia and CVD share pathogenic mechanisms linked to cardiovascular risk factors, and CVD may lower the threshold for cognitive impairment (Song et al., 2020).

According to the Lancet Commission on Dementia Prevention, 12 modifiable risk factors, namely, less education, hypertension, hearing impairment, smoking, obesity, depression, physical inactivity, diabetes, low social contact, excessive alcohol consumption, traumatic brain injury, and air pollution, account for nearly 40% of all dementia cases globally, and this estimate highlights how relatively important potentially modifiable risk factors are in dementia (Livingston et al., 2020). Given that multiple risk factors overlap and may have an additive or synergistic effect (Lee et al., 2019), some risk models that treated incident dementia as the outcome were built, such as Cardiovascular Risk Factors, Aging, and Dementia (CAIDE) risk algorithms (Kivipelto et al., 2006). These models, however, were mainly developed based on predictors that are challenging to obtain in primary prevention, such as apolipoprotein E ℇ4 (ApoE ℇ4), mean corpuscular volume, and peak expiratory flow (Exalto et al., 2014; You et al., 2022). In contrast, CVD risk models have been extensively constructed and applied. The Framingham risk models, which contain health parameters simple to measure, are the most frequently utilized in research and clinical settings (Peloso et al., 2020).

Surprisingly, relevant studies found that CVD risk models are associated with dementia and cognitive decline (Harrison et al., 2014). Using CVD risk models to predict dementia not only impedes the incidence of CVD but also favors putting off the progression of dementia. Harrison conducted a systematic review in 2014 to conclude the association between various CVD risk models and dementia or cognitive impairment (Harrison et al., 2014). In recent years, with the deepening of research, new CVD risk models have been constructed, such as the prediction for atherosclerotic cardiovascular disease (ASCVD) risk in China (China-PAR) (Yang et al., 2016) and Life’s Essential 8 (LE8) (Lloyd-Jones et al., 2022). Furthermore, some scholars have paid more attention to whether CVD risk models can predict dementia and which models have better forecast performance. So, it is necessary to find more evidence and update the results. This review has two objectives: (1) to comprehensively review the association between various CVD risk models and dementia or cognitive decline and (2) to compare the better risk models for predicting dementia and cognitive decline.

## 2. Methods

### 2.1. Search strategy

This systematic review followed the procedures of the Preferred Reporting Items for Systematic Reviews and Meta-analysis (PRISMA) statement (Page et al., 2021). The present study was registered in PROSPERO. Available at: https://www.crd.york.ac.uk/prospero/display_record.php? ID=CRD42023316549. Since Harrison reported related research in 2014 (Harrison et al., 2014), we have systematically reviewed articles from 1 January 2014 until 16 February 2023. PubMed, Web Of Science, PsychINFO, Embase, Cochrane Library, CNKI, Sinomed, and WanFang were searched. Combinations of the following terms were explored: “cognit*,” “dementia,” “Alzheimer’s disease,” “cognitive dysfunction,” “cognitive impair*,” “cognitive decline,” “heart disease risk factors,” “cardiovascular risk,” “cardiovascular risk model,” “cardiovascular risk score,” “cardiovascular health,” “cardiovascular health metrics,” and “Framingham”. The search strategy of PubMed is presented in Table 1.

**Table 1:** PubMed search strategy.

Search formula		Result
#1	[M.H.] = dementia OR cognitive dysfunction OR Alzheimer's disease	221,674
#2	[TIAB] = cognit* OR Dementia OR Alzheimer's disease OR cognitive dysfunction OR MCI OR cognitive impair* OR cognitive decline	608,329
#3	#1 OR #2	693,047
#4	[M.H.] = Heart Disease Risk Factors	5,336
#5	[TIAB] = Heart Disease Risk Factors OR cardiovascular risk OR cardiovascular risk model OR cardiovascular risk score OR cardiovascular health OR cardiovascular health metrics OR Framingham	98,294
#6	#4 OR #5	100,480
#7	#3 AND #6	3,434
#8	#7 AND [2014:2023 (pdat)]	2,249

### 2.2. Inclusion and exclusion criteria

The inclusion criteria for studies were the following: (1) assessed CVD risk models; (2) had access to specific information on cognitive test results (or how they changed); and (3) used a cross-sectional, longitudinal cohort study design. Articles were disqualified if they only examined a single risk factor or included dementia or cognitive impairment data in their risk models. Studies written in languages other than English or Chinese, those with incomplete abstracts, and those lacking full texts were also disqualified.

### 2.3. Data extraction and assessment for study quality

From each eligible study, we extracted data on (1) article information: authors, publication year, and country; (2) cohort information: name of the study, number of participants, percentage of women, mean age, and race/ethnicity; (3) study design: cross-sectional study, longitudinal cohort study (follow-up time); (4) CVD risk models: details of the risk model used and any modifications made; (5) primary outcome (dementia diagnoses, cognitive decline, or cognitive function); and (6) analytical strategy: details on models’ prognostic performance including odds ratio (OR), 95% confidence interval (95% CI), hazard ratio (HR), β coefficients, the area under the receiver operating characteristic curve (AUC), or C-statistic.

### 2.4. Risk of bias assessment

Cohort studies were evaluated for quality using the Newcastle-Ottawa scale (NOS) (Lo et al., 2014). In more detail, the scores of the related items in three domains–four items for the chosen domain, one item for the comparability domain, and three items for the resulting part–were added up to create a total score of the risk of bias. High-quality studies were those with a total score of six or higher. The Agency for Healthcare Research and Quality (AHRQ) provided an 11-item checklist for evaluating the methodological quality of the cross-sectional studies (Newman, 2000). If a question was answered “NO” or “UNCLEAR,” the item received a score of “0” and if the reply was “YES,” the item received a score of “1”. Low-quality articles were given a score of 0–3, moderate quality ones of 4–7, and high-quality ones of 8–11. The prediction model Risk Of Bias ASsessment Tool (PROBAST) includes 20 signaling questions across four domains: participants, predictors, outcome, and analysis (Moons et al., 2019). It is used to assess a prediction model’s risk of bias and applicability concerns. It can be downloaded from www.probast.org.

### 2.5. Data synthesis

Due to the variation among studies, including variations in the variables included in the CVD risk models, the small number of studies using the same models, and even variations in adjusted variables across studies using the same models, as well as differences in cognitive outcomes and variations in the reporting of effect sizes, synthesis with meta-analysis is not possible.

## 3. Results

### 3.1. Description of included studies

**Figure 1:**
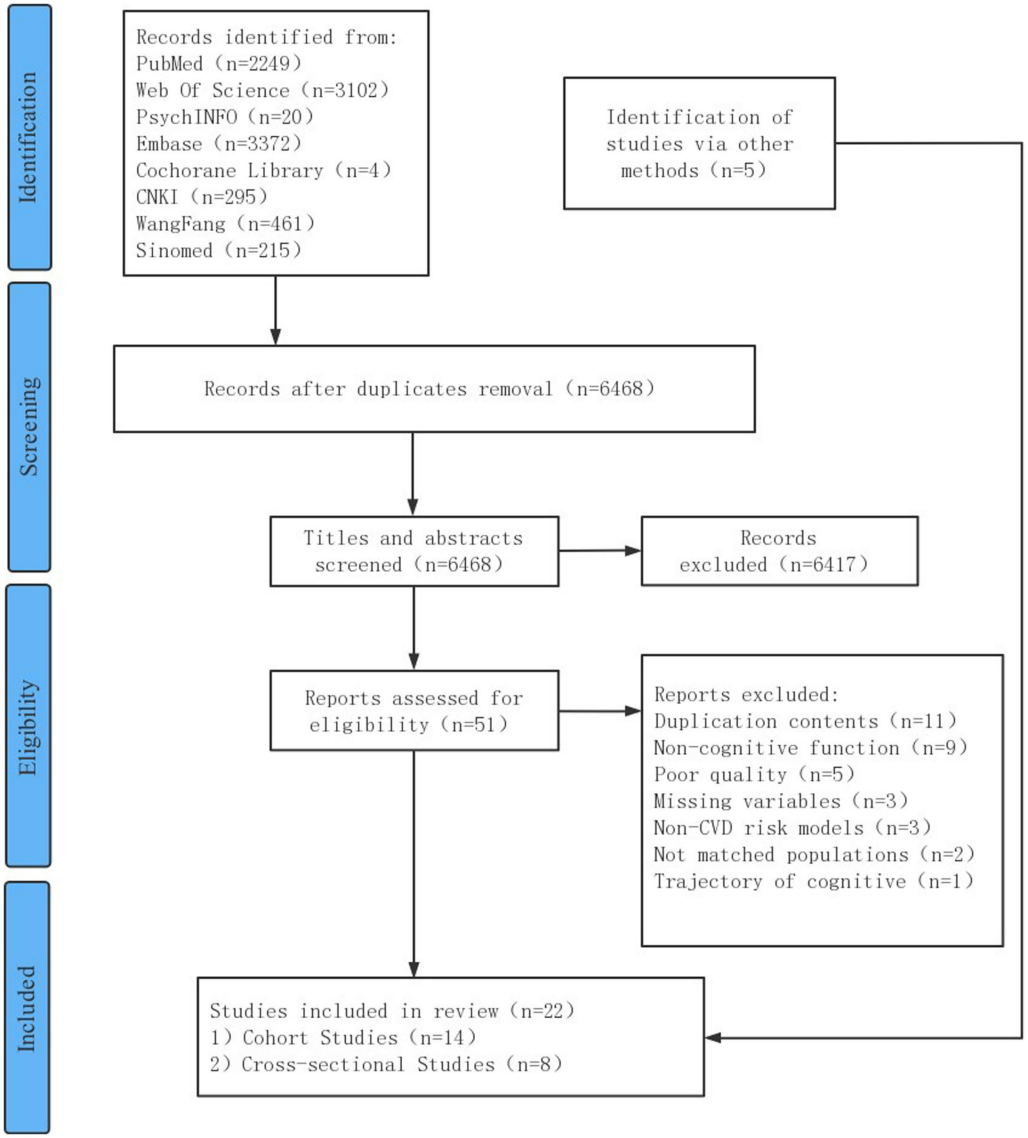
PRISMA 2020 flow diagram presenting study screening and selection process.

The search yielded a total of 9,718 articles. Of these, 3,250 were duplicates, and 6,417 were irrelevant after title and abstract reviews. A total of 51 articles underwent full-text screening and 34 were excluded due to unmet criteria. Other 5 extra studies were obtained from the references of the included articles and the citation retrieval of related systematic reviews, and 22 full-text articles, including 14 cohort studies and 8 cross-sectional studies, were included. Figure 1 summarizes the study selection process.

The included research was conducted in 10 different countries. The most significant articles came from the United States, China, Italy, France, Thailand, the Republic of Korea, and the United Kingdom. A total of 19 studies were derived from datasets based on data from a population-based cohort. Most studies used the National Alzheimer Coordinating Center (NACC) database (Jefferson et al., 2015; Viswanathan et al., 2015; Pelcher et al., 2020). Sample sizes ranged from 300 to 429,033 and were racially diverse as they included White people, Black people, Hispanic/Latino (H/L) people, and non-H/L people. The mean age at baseline ranged from 45 to 79.5 years. In cohort studies, the follow-up duration ranged from 1 to 26.2 years. Detailed information on the study cohort, sample traits, CVD risk models, cognitive function or dementia assessments, and model details are presented in Table 2.

**Table 2:** Summary characteristics of the included studies.

First author/publication year	Country	Cohort	Sampling				CVD risk model	Outcome	Study design	Follow-up duration (year)	Effect values
			*N*	Mean age	Gender (Females %)	Multiethnic					
Zheng et al. (2022)	China	UK Biobank cohort	429,033	57.1	53.8%	N	SCORE2 SCORE CAIDE	Dementia (AD, VD)	Cohort	12.8	C-indices: 0.750 (0.745 to 0.755)
Buawangpong et al. (2022)	Thailand	/	421	63.39	64%	N	TCVR	MCI	Cohort	7	AuROC: 0.58–0.61
McGrath et al. (2022)	United States	FHS Original and Offspring cohort	4,899 2,386	55 80	57.2% 62.1%	N	FSRP (rFSRP) PGRS	Dementia	Cohort	10	HR: 1.160 (1.06–1.26)
Tin et al. (2022)	United States	ARIC	8,823EA 2,738AA	54.3EA 53.5AA	53.1%EA 63.1%AA	N	LS7	Dementia	Cohort	26.2	HR (EA): 1.440 (1.37–1.51) HR (AA): 1.260 (1.16–1.36)
Ji et al. (2022)	China	CHARLS	6,402	57.8	49.0%	N	FGCRS	Cognitive function	Cohort	6.9	β: −0.001 (−0.006 to −0.004)
Schaich et al. (2022)	United States	MESA	4,392	60.1	53%	Y	CAIDE FSRP ASCVD-PCE	Cognitive decline	Cohort	16	FSRP: HR: 1.500 (1.30–1.74); AUC: 0.66 ASCVD-PCE: HR: 1.620 (1.40–1.88); AUC: 0.65
Song et al. (2020)	China	MAP (northeastern Illinois)	1,588	79.5	76%	N	FGCRS	Cognitive function MCI Dementia	Cohort	21	β: −0.019 (−0.035 to −0.003)
Rundek et al. (2020)	United States	NOMAS	1,290	64	60%	Y	CAIDE GVRS	Cognitive decline	Cohort	6	SE: −0.247
Samieri et al. (2018)	France	Three city Study	6,626	73.3	63.4%	N	LS7	Cognitive decline	Cohort	12–16	HR: 0.920 (0.89–0.96)
Viticchi et al. (2017)	Italy	Amnestic MCI	385	72.29	49.4%	N	FCRP	AD	Cohort	1	AUC: 0.682 (0.577–0.786)
Harrison et al. (2017)	United Kingdom	Newcastle 85+ Study Leiden 85-plus Study LiLACS NZ Study	616 444 396	85+ 85-plus	60.1% 65.4% 52%	N	FSRP CAIDE Oxi-inflammatory load	Cognitive function	Cohort	60 months annually for 5 annually for 3	HR: 1.460 (1.08–1.98)
Viswanathan et al. (2015)	United States	NACC	2,975	72.1	66.1%	N	rFSRP	Cognitive function	Cohort	3.18 ± 1.35	β: −0.059 (−0.188 to −0.069)
Jefferson et al. (2015)	United States	NACC	3,117MCI 6,603NC	74 72	56% 68%	N	FSRP	Dementia MCI Cognitive function	Cohort	2.2 ± 2.2MCI 3.0 ± 2.5NC	β: −0.01 (−0.03–0.02)
Thacker et al. (2014)	United States	REGARDS	17,761	45+	55%	Y	LS7	Cognitive function	Cohort	A mean of 4	HR: 0.630 (0.51–0.79)
Wei et al. (2022)	United States	NHANES	2,585	≥60	54%	N	LS7	Cognitive function	Cross-sectional	/	β: 0.05 (0.02–0.07)
Mun et al. (2023)	Republic of Korea	NHIS	8,600	69.74	60.5%	N	KRS	Cognitive decline	Cross-sectional	/	OR: 1.339 (1.034–1.734)
Jeon et al. (2021)	Republic of Korea	CMERC	2,622	57.2	68.3%	N	CANHEART health index	Cognitive function	Cross-sectional	/	β: 1.99 (1.01–3.92)
Bao-Shan et al. (2021)	China	/	300	61.93	60%	N	China-PAR	Cognitive function	Cross-sectional	/	OR: 2.586 (1.023–6.533)
Torres et al. (2020)	United States	FRONTIER	541	61.6	68.9%	N	CAIDE FRS WHICAP	Cognitive function	Cross-sectional	/	β (FRS): −0.08 β (WHICAP): −0.04
Pelcher et al. (2020)	United States	NACC-UDS	19,309	72.84	56.9%	N	rFSRP	Dementia MCI Cognitive function	Cross-sectional	/	β: 0.02 (0.001–0.01)
Tarraf et al. (2020)	United States	HCHS/SOL	7,650	56	55.6%	N	FCRS GVRS	Cognitive function	Cross-sectional	/	β (FGCRS): −0.019 β (GVRS): −0.042
Badran et al. (2019)	United Kingdom	/	346	57	54%	N	Framingham Vascular Age	Cognitive function	Cross-sectional	/	β: −0.005

SCORE, Systematic COronary Risk Evaluation; CAIDE, Cardiovascular Risk Factors, Aging, and Dementia; AUC, area under the receiver operating characteristic curve; TCVR, Thai Cardiovascular Risk score; MCI, mild cognitive impairment; FHS, Framingham Heart Study; PGRS, a polygenic risk score; NHIS, National Health Insurance Service; ARIC, Atherosclerosis Risk in Communities; LS7, Life’s Simple 7; CAIDE, Cardiovascular Risk Factors, Aging, and Incidence of Dementia; AD, Alzheimer’s disease; VD, Vascular disease; FSRP, Framingham Stroke Risk Profile; ASCVD-PCE, atherosclerotic cardiovascular disease pooled cohort equation; MAP, The Rush Memory and Aging Project; FGCRS, The Framingham General Cardiovascular Risk Score; NOMAS, Northern Manhattan Study; NACC, National Alzheimer Coordinating Center; NC, normal cognitive; REGARDS, Reasons for Geographic And Racial Differences in Stroke; NHANES, National Health and Nutrition Examination Survey; KPS, Korean coronary heart disease risk score; OR, odds ratio; CMERC, The Cardiovascular and Metabolic Diseases Etiology Research Center study; CANHEART, Cardiovascular Health in Ambulatory Care Research Team; China-PAR, Prediction for ASCVD Risk in China; FRONTIER, Facing Rural Obstacles to Healthcare Now Through Intervention, Education & Research; FRS, Framingham Risk Score; WHICAP, Washington Heights-Inwood Community Aging Project Risk Score; NACC-UDS, National Alzheimer’s Coordinating Center Uniform Data Set; rFSRP, revised Framingham Stroke Risk Profile; HCHS/SOL, Hispanic Community Health Study/Study of Latinos; FCRS, Framingham Cardiovascular Risk Score; GVRS, Global Vascular Risk Score; N, No; Y, Yes; U, Unclear; FSRP, Framingham Stroke Risk Profile; CHARLS, the China Health and Retirement Longitudinal Study; EA, European American; AA, African American; HR, Hazard Ratio; β, β coefficients; SE, standard error.

### 3.2. Quality of the included studies

After the studies’ quality assessment, the 14 cohort studies were rated as good quality, and the 8 cross-sectional studies were rated moderate to high. The deduction items are concentrated in 6 and 9–11. The key concern is that several cardiovascular parameters (physical activity and diet) were dependent on self-reports. In addition, some studies substituted similar variables because existing cohorts did not contain the desired variables. For example, Washington Heights-Inwood Community Aging Project Risk Score (WHICAP) studies used Body Mass Index (BMI) instead of the waist-to-hip ratio (Torres et al., 2020). The quality assessment of the reviewed studies is shown in Tables 3, 4.

**Table 3:** The quality assessments of the cohort studies.

Cohort star template(NOS)			
Study author (publication date, country)	Selection	Comparability	Outcome
Zheng et al. (2022), China	✭✭✭✭	✭✭	✭✭✭
Buawangpong et al. (2022), Thailand	✭✭✭✭	✭✭	✭✭
McGrath et al. (2022), United States	✭✭✭✭	✭✭	✭✭✭
Tin et al. (2022), United States	✭✭✭✭	✭✭	✭✭✭
Ji et al. (2022), China	✭✭✭✭	✭✭	✭✭✭
Schaich et al. (2022), United States	✭✭✭✭	✭✭	✭✭✭
Song et al. (2020), China	✭✭✭✭	✭✭	✭✭✭
Rundek et al. (2020), United States	✭✭✭✭	✭✭	✭✭✭
Samieri et al. (2018), France	✭✭✭✭	✭✭	✭✭✭
Viticchi et al. (2017), Italy	✭✭✭✭	✭✭	✭✭
Harrison et al. (2017), United Kingdom	✭✭✭✭	✭✭	✭✭✭
Viswanathan et al. (2015), United States	✭✭✭✭	✭✭	✭✭
Jefferson et al. (2015), United States	✭✭✭✭	✭✭	✭✭✭
Thacker et al. (2014), United States	✭✭✭✭	✭✭	✭✭

**Table 4:** The quality assessments of cross-sectional studies.

Cross-sectional study quality(AHRQ)												
Study author (publication date, country)/items	1	2	3	4	5	6	7	8	9	10	11	Quality score
Wei et al. (2022), United States	Y	Y	Y	Y	Y	U	Y	Y	U	Y	U	8
Mun et al. (2023), Republic of Korea	Y	Y	Y	Y	N	U	Y	Y	U	Y	Y	8
Jeon et al. (2021), Republic of Korea	Y	Y	Y	Y	N	N	Y	Y	U	Y	U	7
Bao-Shan et al. (2021), China	Y	Y	Y	Y	N	U	Y	Y	U	N	U	6
Torres et al. (2020), United States	Y	Y	N	Y	N	U	Y	Y	U	Y	U	6
Pelcher et al. (2020), United States	Y	Y	Y	Y	N	U	Y	Y	U	Y	U	7
Tarraf et al. (2020), United States	Y	Y	N	Y	Y	U	Y	Y	U	N	U	6
Badran et al. (2019), United Kingdom	Y	Y	N	Y	N	N	Y	Y	U	Y	U	6

1. Define the source of information (survey, record review); 2. List inclusion and exclusion criteria for exposed and unexposed subjects (cases and controls) or refer to previous publications; 3. Indicate time used for identifying patients; 4. Indicate whether or not subjects were consecutive if not population-based; 5. Indicate if evaluators of subjective study components were masked to other aspects of the status of the participants; 6. Describe any assessments undertaken for quality assurance purposes(e.g., test/retest of primary outcome measurements); 7. Explain any patient exclusions from analysis; 8. Describe how confounding was assessed and controlled; 9. If applicable, explain how missing data were handled in the analysis; 10. Summarize patient response rates and completeness of data collection; 11. Clarify what follow-up, if any, was expected and the percentage of patients for which incomplete data or follow-up was obtained. Y, Yes; N, No; U, Unclear.

### 3.3. Quality of the included CVD risk models

A total of 22 studies described 15 CVD prediction models (Kannel et al., 1976; D’Agostino et al., 1994, 2008; Conroy et al., 2003; Sacco et al., 2009; Lloyd-Jones et al., 2010; Reitz et al., 2010; Goff et al., 2014; Jee et al., 2014; Maclagan et al., 2014; Tsao and Vasan, 2015; Yang et al., 2016; Dufouil et al., 2017; Hageman et al., 2021; Buawangpong et al., 2022). Among them were two models without evaluation (Lloyd-Jones et al., 2010; Maclagan et al., 2014). Life’s Simple 7 (LS7) was defined as the concept and determining of the metrics done by the Goals and Metrics Committee of the Strategic Planning Task Force of the American Heart Association (AHA) (Lloyd-Jones et al., 2010). Furthermore, the Cardiovascular Health in Ambulatory Care Research Team (CANHEART) health index was a modified version of the AHA’s definition of ideal cardiovascular health (CVH) for Canadians (Maclagan et al., 2014). Besides, WHICAP claimed good prediction performance, with no specific values discovered (Reitz et al., 2010). Others found AUC or C-statistics ranging from 0.51 to 0.86. Except for the Thai Cardiovascular Risk score (TCVR) (Buawangpong et al., 2022), most of the models demonstrated good to acceptable discrimination (Receiver Operator Characteristic Curve>0.7). No studies exhibited a low risk of bias owing to insufficient analytical domain reporting (Tables 5, 6). The explanation could be that the CVD risk models were built and verified before the PROBAST report was proposed, and there needed to be a uniform and standard reporting checklist, resulting in a low assessment score. In contrast, its predictive performance was quite good, particularly for the Framingham series models.

**Table 5:** The analysis of four domains of CVD risk models.

Included Study	**①**	**②**	**③**	**④**	**⑤**	**⑥**	**⑦**	**⑧**	**⑨**	**⑩**	⑪	⑫	⑬	⑭	⑮	⑯	⑰	⑱	⑲	⑳	Performance
FSRP	Y	Y	Y	Y	Y	Y	Y	Y	Y	NI	Y	Y	Y	Y	NI	Y	NI	Y	Y	Y	For men: Year Probability of Stroke: 9.6% For women: 10-Year Probability of Stroke: 6.5%
rFSRP	Y	Y	Y	Y	Y	Y	Y	Y	Y	NI	Y	Y	N	Y	Y	Y	NI	Y	N	Y	For men: Calibration chi-squares: 64.0/12.1 For women: Calibration chi-squares: 42.5/4.1
FRS	Y	Y	Y	Y	Y	Y	Y	Y	Y	NI	Y	Y	N	Y	NI	Y	NI	N	N	Y	For men: C-Index =0.74 For women: C-Index =0.77
FCRS	Y	Y	Y	Y	Y	Y	Y	Y	Y	NI	Y	Y	NI	Y	Y	Y	NI	Y	Y	Y	C-Index: 0.756 (95%CI: 0.739–0.773)
Framingham Vascular Age	Y	Y	Y	Y	Y	Y	Y	Y	Y	NI	Y	Y	NI	Y	Y	Y	NI	Y	Y	Y	C-Index: 0.756 (95%CI: 0.739–0.773)
KPS	Y	Y	Y	Y	Y	Y	Y	Y	Y	NI	Y	Y	NI	Y	Y	Y	NI	Y	N	Y	For men: AUC =0.764 For women: AUC =0.815
SCORE	Y	Y	Y	Y	Y	Y	Y	Y	Y	NI	Y	Y	NI	Y	NI	Y	NI	Y	Y	Y	ROC: 0.71–0.84
SCORE2	Y	Y	Y	Y	Y	Y	Y	Y	Y	NI	Y	Y	NI	Y	Y	Y	NI	Y	N	Y	C-Index: 0.81 (0.76–0.86)
TCVR	Y	Y	Y	PY	Y	Y	Y	Y	Y	NI	PY	N	Y	Y	Y	Y	NI	Y	Y	Y	AuROC = 0.58–0.61
China-PAR	Y	Y	Y	PY	Y	Y	Y	Y	Y	NI	Y	Y	Y	Y	Y	Y	NI	Y	NI	Y	For men: C-Index: 0.794 (95%CI: 0.775–0.814) For women: C-Index: 0.811 (95%CI: 0.787–0.835)
WHICAP	Y	Y	Y	PY	Y	Y	Y	Y	Y	NI	PY	Y	NI	N	Y	Y	NI	N	N	Y	NI
GVRS	Y	Y	Y	PY	Y	Y	Y	Y	Y	NI	Y	Y	Y	Y	NI	Y	NI	Y	Y	Y	ROC = 0.747
ASCVD-PCE	Y	Y	Y	PY	Y	Y	Y	Y	Y	NI	Y	Y	NI	Y	NI	Y	NI	Y	N	Y	C-Index: 0.713–0.818

Y, Yes; PY, Probably Yes; N, No; PN, Probably No; NI, No Information; CI, Confidence Interval; AUC, Area Under the Receiver Operating Characteristic Curve; ROC, Receiver Operator Characteristic Curve ①Were appropriate data sources used, e.g., cohort, randomized controlled trial, or nested case–control study data? ②Were all inclusions and exclusions of participants appropriate? ③Were predictors defined and assessed in a similar way for all participants? ④Were predictor assessments made without knowledge of outcome data? ⑤Are all predictors available at the time the model is intended to be used? ⑥Was the outcome determined appropriately? ⑦Was a prespecified or standard outcome definition used? ⑧Were predictors excluded from the outcome definition? ⑨Was the outcome defined and determined in a similar way for all participants? ⑩Was the outcome determined without knowledge of predictor information? ⑪Was the time interval between predictor assessment and outcome determination appropriate? ⑫Were there a reasonable number of participants with the outcome? ⑬Were continuous and categorical predictors handled appropriately? ⑭Were all enrolled participants included in the analysis? ⑮Were participants with missing data handled appropriately? ⑯Was selection of predictors based on univariable analysis avoided? (Model development studies only) ⑰Were complexities in the data (e.g., censoring, competing risks, sampling of control participants) accounted for appropriately? ⑱Were relevant model performance measures evaluated appropriately? ⑲Were model overfitting and optimism in model performance accounted for? (Model development studies only) ⑳Do predictors and their assigned weights in the final model correspond to the results from the reported multivariable analysis? (Model development studies only).

**Table 6:** The PROBAST results of CVD risk models.

Included Study	ROB				Applicability			Overall	
	Participants	Predictors	Outcome	Analysis	Participants	Predictors	Outcome	ROB	Applicability
FSRP	+	+	?	?	+	+	+	?	+
rFSRP	+	+	?	−	+	+	+	−	+
FRS	+	+	?	−	+	+	+	−	+
FCRS	+	+	?	?	+	+	+	?	+
Framingham vascular age	+	+	?	?	+	+	+	?	+
KPS	+	+	?	−	+	+	+	−	+
SCORE	+	+	?	?	+	+	+	?	+
SCORE2	+	+	?	−	+	+	+	−	+
TCVR	+	+	?	−	+	+	?	−	?
China-PAR	+	+	?	?	+	+	+	?	+
WHICAP	+	+	?	−	+	+	?	−	?
GVRS	+	+	?	?	+	+	+	?	+
ASCVD-PCE	+	+	?	−	+	+	+	−	+

PROBAST, Prediction model Risk Of Bias ASsessment Tool; ROB, risk of bias. * + indicates low ROB/low concern regarding applicability; − indicates high ROB/high concern regarding applicability; and? indicates unclear ROB/unclear concern regarding applicability.

### 3.4. The different CVD risk models and dementia or cognitive decline

Framingham risk models and CAIDE, and other nine CVD risk models were used to predict dementia or cognitive decline, namely, the Korean Coronary Heart Disease Risk score (KPS), Systematic COronary Risk Evaluation (SCORE), SCORE2, atherosclerotic cardiovascular disease pooled cohort equation (ASCVD-PCE), TCVR, CANHEART, Global Vascular Risk score (GVRS), China-PAR, and LS7. Generally, the included CVD risk models had 5 to 15 factors. GVRS had the most number of factors, whereas SCORE had the least (Conroy et al., 2003; Sacco et al., 2009). Except for CANHEART and LS7, other models all included sex, age (age-stratified), systolic blood pressure (SBP), and smoking habits. Notably, race was taken into consideration by ASCVD-PCE, GVRS, and WHICAP (Rundek et al., 2020; Tarraf et al., 2020; Torres et al., 2020; Schaich et al., 2022). WHICAP is a simple summary risk score for predicting Alzheimer’s disease (AD) in older adults (Reitz et al., 2010). Given that its construction was based on cardiovascular risk factors, we decided that WHICAP could be included. All of the risk factors can be divided into traditional factors, behavioral factors, and anthropometric factors, including demographic data (age and sex), laboratory data (BP, blood glucose, and APOE ℇ4), questionnaire data (physical activity and dietary habits) and self-report data (physical activity, dietary habits, and smoking habits). Please see Tables 7, 8 for more details.

**Table 7:** The details of Framingham risk models.

Framingham risk models		
CVD risk model	Population	Variables included
FSRP	White American	Age, sex, SBP, use of antihypertension therapy, DM, CVD, AF, left ventricular hypertrophy, and cigarette smoking
rFSRP	White American	Age, sex, SBP, current smoking status, prevalent CVD, prevalent or past AF, prevalent or past DM, and antihypertensive medication use
FRS	White American	Age, gender, current smoking status, TC, HDL-c, SBP, and use of blood pressure medications
FCRS	White American	Age, sex, TC, HDL-c, SBP, and blood pressure treatment, smoking, and DM
Framingham Vascular Age	White American	Age, gender, SBP, antihypertensive medication, smoking, Type 2 DM, and BMI

FSRP, Framingham Stroke Risk Profile; FRS, Framingham Risk Score; FCRS, Framingham Cardiovascular Risk Score; rFSRP, revised Framingham Stroke Risk Profile; SBP, Systolic Blood Pressure; TC, Total Cholesterol; LDL_c, Low-density Lipoprotein Cholesterol; HDL_c, High-density Lipoprotein Cholesterol; BMI, Body Mass Index; AF, atrial fibrillation; CVD, Cardiovascular Disease; DM, Diabetes Mellitus.

**Table 8:** The details of specific CVD risk models.

Specific CVD risk models		
CVD risk model	Population	Variables included
KPS	Korean	Sex, age, SBP, DBP, TC, HDL-c, smoking status, and diabetes history
SCORE	European	Sex, age, smoking, SBP, TC, or cholesterol/HDL-c ratio
SCORE2	European	Age, current smoking, SBP, diabetes, TC, and HDL-c
TCVR	Thai	Age, gender, DM, SBP, WC, height, TC, HDL-c, and LDL-c The TCVR can be calculated using six models: age, gender, DM, smoking status, SBP, and WC; age, gender, DM, smoking status, SBP, WC, and height; age, gender, DM, smoking status, SBP, and TC; age, gender, DM, smoking status, SBP, TC, and HDL-c; age, gender, DM, smoking status, SBP, HDL, and LDL-c; age, gender, DM, smoking status, SBP, and LDL-c.
CANHEART	Canadian	Smoking status, physical activity, healthy diet, obesity, DM, and hypertension history
China-PAR	Chinese	Age, treated or untreated SBP, TC, HDL-C, current smoking, DM, WC, geographic region, urbanization, and family history of ASCVD
LS7	General	Smoking status, BMI, physical activity, diet, TC, blood pressure, and fasting blood glucose
WHICAP	34.2% White population 30.6% Black population 33.3% Hispanic/Latino population	Sex, age, presence of DM or hypertension, current smoking status, low HDL, waist-to-hip ratio (BMI), education, ethnicity, and APOE ε4 allele status
GVRS	52.7% Hispanic population 24.9% African American population 19.9% White population	Age, gender, African American, Hispanic Ethnicity, waist, alcohol, former or current smoker, moderate or moderate-to-heavy activity, SBP, DBP, antihypertensive medication, peripheral vascular disease, fasting blood glucose, HDL-c, and TC
ASCVD-PCE	Asian and multiethnic populations that include Hispanic people	Age, sex, SBP, TC, HDL-c, antihypertensive medication use, DM, current smoker, and race

KPS, Korean coronary heart disease risk score; SCORE, Systematic Coronary Risk Evaluation; CAIDE, Cardiovascular Risk Factors, Aging, and Incidence of Dementia; ASCVD_PCE, atherosclerotic cardiovascular disease pooled cohort equation; TCVR, Thai Cardiovascular Risk score; CANHEART, Cardiovascular Health in Ambulatory Care Research Team; WHICAP, Washington Heights_Inwood Community Aging Project Risk Score; GVRS, Global Vascular Risk Score; China-PAR, Prediction for ASCVD Risk in China; LS7, Life’s Simple 7; SBP, Systolic Blood Pressure; DBP, Diastolic Blood Pressure; TC, Total Cholesterol; LDL_c, Low-density Lipoprotein Cholesterol; HDL_c, High-density Lipoprotein Cholesterol; BMI, Body Mass Index; DM, Diabetes Mellitus; WC, White Cell; ASCVD, Atherosclerotic Cardiovascular Disease.

Compared with previous research, more studies focused on cognitive function than dementia (Harrison et al., 2014). Dementia was further diagnosed in different subcategories, including AD and vascular disease (VD) (Zheng et al., 2022). There were two ways for included studies to assess outcomes: one was cognitive diagnoses, including International Classification of Diseases 9 or 10 (ICD-9, ICD-10), an expert committee, clinical examination, or Clinical Dementia Rating (CDR), and the other way was neuropsychological assessments including Mini-Mental State Examination (MMSE), Montreal Cognitive Assessment (MOCA), or domain-specific tests. Furthermore, global cognitive function could be acquired by calculating Z scores.

Except for one study (Jeon et al., 2021), the various risk models were found to be significantly connected with a higher chance of dementia in the future. In this study, CVH levels did not correlate with early cognitive decline in the Korean study population. The validity of the CANHEART model used to measure CVH was not established for the Korean population because it was initially developed by Canadian academics for the Canadian population. In addition, the CANHEART model used in Canadian samples for predicting cognitive decline was unclear.

The results were ambiguous. AUC was the prognostic performance metric in only four studies (Viticchi et al., 2017; Buawangpong et al., 2022; Schaich et al., 2022; Zheng et al., 2022). More studies just analyzed β efficiency. According to the individual risk factor profiles in midlife, the CAIDE risk score was explicitly created as an easy method to forecast the likelihood of dementia in the future (Kivipelto et al., 2006). With an initial C-index of 0.78 and a repeated C-index of 0.75 following external validation, the score accurately predicted dementia (Exalto et al., 2014).

### 3.5. The Framingham CVD risk models and dementia or cognitive decline

A total of 12 studies used the Framingham risk model: five studies used the Framingham General Cardiovascular Risk Score (FGCRS) (Viticchi et al., 2017; Song et al., 2020; Tarraf et al., 2020; Torres et al., 2020; Ji et al., 2022), three studies used the Framingham Stroke Risk Profile (FSRP) (Jefferson et al., 2015; Harrison et al., 2017; Schaich et al., 2022), while three studies used the revised Framingham Stroke Risk Profile (rFSRP) (Viswanathan et al., 2015; Pelcher et al., 2020; McGrath et al., 2022), which eliminates left ventricular hypertrophy (LVEF), and one study used Framingham Vascular Age (Badran et al., 2019). All studies found higher Framingham risk scores associated with an increased risk of subsequent cognitive decline or impairment. Which cognitive abilities were linked to a higher risk of CVD or stroke varied among research due to various measures of cognitive function, sample sizes, or statistical performance.

### 3.6. Specific CVD risk models and dementia or cognitive decline

Except for Framingham models, we concluded that nine specific CVD risk models may be associated with dementia and cognitive decline. Five CVD risk models were designed for a single country (Bao-Shan et al., 2021; Jeon et al., 2021; Buawangpong et al., 2022; Zheng et al., 2022; Mun et al., 2023). KPS, TCVR, the China-PAR, SCORE2, and the CANHEART health index were developed to measure cardiovascular risk among Korean, Thai, Chinese, European, and Canadian populations.

Four risk models were for the multiethnic cohort. A longitudinal cohort of non-Hispanic White people and African Americans served as the basis for the ASCVD-PCE (Schaich et al., 2022) and has since been validated for predicting atherosclerotic CVD risk in Asian and multiethnic populations, including Hispanics. A diverse group with almost equal representations of White people (34.2%), Black people (30.6%), and H/L (33.3%) participants created the WHICAP summary risk score to predict AD in older adults based on their vascular profiles associated with late-onset AD (Torres et al., 2020). GVRS is a model for assessing global vascular risk developed in the Northern Manhattan Study (NOMAS) that incorporates traditional, behavioral, and anthropometric risk factors and uses continuous variables (Rundek et al., 2020; Tarraf et al., 2020). A simple tool, LS7 only has seven modified factors, including four behavioral and three biological factors, similar to CANHEART (Lloyd-Jones et al., 2010). The AHA first suggested this metric for keeping CVH and has since been advised for brain health (Gorelick et al., 2017).

Other than CANHEART, specific CVD risk models were associated with a lower risk for dementia or cognitive decline (Covello et al., 2021; Jeon et al., 2021; Speh et al., 2021). CVD risk models from multiethnic cohorts were proven to predict dementia or cognitive decline in diverse race populations, such as White, H/L, Black, or Asian. Specifically, an article reported the association between QRISK and cognitive function, but we did not report it due to its poor quality (Hsu et al., 2015).

Only SCORE2, FSRP, ASCVD-PCE, and FCRP reported the models’ performance for dementia (Viticchi et al., 2017; Schaich et al., 2022; Zheng et al., 2022). The AUC or C-statistics of these models ranged from 0.65 to 0.75. Additionally, the C-indices of SCORE2 risk could distinguish between incident all-cause dementia, AD, VD, and all-cause death (Zheng et al., 2022).

### 3.7. Comparing the association between different CVD risk models and dementia or cognitive decline

Seven studies compared the predictive performance of two or three risk models (Harrison et al., 2017; Rundek et al., 2020; Tarraf et al., 2020; McGrath et al., 2022; Schaich et al., 2022; Zheng et al., 2022). CAIDE, as a reference substance, was used for five articles. Some studies reported that CAIDE is better for predicting dementia. For instance, one study that evaluated FSRP, ASCVD-PCE, and CAIDE concluded that CAIDE had more robust relationships with cognitive performance than FSRP and ASCVD-PCE but that ASCVD-PCE’s associations with the Digit Symbol Coding (DSC) and Digit Span (DS) were comparable to CAIDE’s (*β* = −0.57 and − 0.21, respectively) (Schaich et al., 2022).

Other studies showed that CAIDE had an inferior association with dementia or cognitive function compared with other risk models, such as SCOPE2, WHICAP, or GVRS. One study that compared SCORE2 with SCORE and CAIDE showed that the C-index of SCORE2 risk for discriminating against all-cause dementia was 0.750, which was significantly higher than the C-indices of SCORE and CAIDE risk (SCORE2 vs. SCORE: 0.014; SCORE2 vs. CAIDE: 0.093). The study further found that SCORE2 risk significantly improved the ability to distinguish between AD and VD (Zheng et al., 2022). Two studies showed that higher GVRS presented stronger associations with lower cognitive function than the FCRS or CAIDE (Rundek et al., 2020; Tarraf et al., 2020).

## 4. Discussion

This systematic review synthesizes the current trends regarding the association between different CVD risk models and incident dementia or cognitive decline. We updated Harrison’s systematic review and searched the articles from 2014 to 2023 (Harrison et al., 2014). As a result, 22 studies were included, and 15 CVD risk models were included; besides the Framingham series of models (FSRP, rFSRP, FRS, FGCRS, and Framingham vascular age), there were also specific CVD risk models, namely, KPS, TCVR, China-PAR, SCORE1, SCORE2, CANHEART, ASCVD-PCE, WHICAP, GVRS, and LS7. Our results show that higher CVD risk scores were associated with an increased risk of subsequent dementia or cognitive impairment. Furthermore, compared to the traditional dementia predicting model, multiethnic CVD risk models had a higher connection with cognitive tests.

Regarding the target outcome, only CAIDE was built to predict incident dementia, while other risk models treated fatal or non-fatal CVD events as outcomes. However, more studies found that CVD risk models were associated with dementia and cognitive impairment, and even some articles that compared CAIDE with CVD risk models proved that some CVD risk models’ dementia-predicting performance may be similar to or better than CAIDE. Kaffashian et al. (2013) discovered that the Framingham risk scores may be more accurate at predicting future cognitive decline than the CAIDE score by contrasting the FGCRS and FSPR with the CAIDE risk score (Kaffashian et al., 2013). One study compared CAIDE, FSRP, and ASCVD-PCE with exceptional cognitive performance in 4,392 multiethnic studies of participants with atherosclerosis and found that CAIDE had stronger associations with cognitive abilities performance than the FSRP and ASCVD-PCE. However, associations of ASCVD-PCE with processing speed and working memory were similar to CAIDE (Schaich et al., 2022). It is clear that Framingham risk models, consisting of cardiovascular factors, are more appropriate for predicting cognitive impairment. Similar to how SCORE2, WHICAP, and GVRS were created to address a variety of incident CVD risk variables, the findings of this study showed that those models could also predict incident dementia better than the CAIDE risk algorithm (Rundek et al., 2020; Tarraf et al., 2020; Torres et al., 2020; Zheng et al., 2022).

Higher scores from the CVD risk models have been linked to an increased risk of cognitive deterioration or dementia in many studies. Still, when specific cognitive domains have been examined, the results have been mixed regarding which cognitive domains have higher cardiovascular risk (Harrison et al., 2014; Wu et al., 2023). Notably, the identified studies had different primary cognitive outcome measures, which impede inferences regarding which vascular risk scores are the most responsive to specific cognitive domains. Many cognitive tests were used to identify incident dementia or cognitive decline, including verbal fluency, short-term and long-term memory, computation, processing speed, and exclusion function. Some studies reported that FGCRS and GVRS were associated with episodic memory, while others proved that no association was observed for episodic memory when the rFSRP was used (Viswanathan et al., 2015). However, in the NACC cohort, after controlling for age, FSRP independently contributed to a decline in processing speed (Jefferson et al., 2015). The variation of cohort characteristics (e.g., ethnicity and education level), methodological variances, follow-up time, and cognitive assessment instruments may all contribute to the differences in the results.

Traditional risk factors for CVD, such as hypertension, diabetes, dyslipidemia, obesity, and smoking, have been linked to a rapid loss of cognitive function. Moreover, most studies that showed an association between CVD risk models and worse cognitive function were focused on a single population, such as White people, whereas there were fewer studies conducted within non-White populations, such as H/L. It has been shown that race/ethnicity, as a critical element, modified associations (Tarraf et al., 2020; Torres et al., 2020; Schaich et al., 2022). Schaich et al. (2022) found that associations between CAIDE and dementia were more significant in African Americans and Hispanics than in White individuals (difference in *β* = 0.69 and 1.67, respectively). CVD risk models, such as the multiethnic GVRS, that are tailored to specific risks based on racial/ethnic background and that can offer significant insight into cognitive risk are practical to use in primary care settings as opposed to FCRP, which ignores race (Tarraf et al., 2020).

Better than GVRS, which contains too many factors, LS7 and CANHEART emphasize modifiable risk factors more than other risk models and may have more immediate implications for health promotion and disease prevention. As Canadian researchers originally created the CANHEART for the Canadian population, it does not have the TC constraint and its validity has not been demonstrated for the Korean population. Unlike CANHEART, LS7 was proposed by the AHA and includes seven modifiable cardiovascular risk factors. The relationships between the seven individual components and cognitive outcomes have been extensively researched. Nonetheless, the results were mixed since the connections between vascular risk factors and dementia are frequently complex, non-linear, and age-dependent. It is generally accepted that midlife CVH tends to have a linear association with late-life dementia risk.

In contrast, a J-shaped association was seen between the late-life CVH score and dementia, according to a systematic review and meta-analysis (Wu et al., 2023). Furthermore, in 2022, the AHA updated LS7 to LE8, optimizing the scoring algorithm and adding sleep information (Lloyd-Jones et al., 2022). Following the AHA’s CVH recommendations and keeping CVH at its best will significantly lower the risk of developing dementia in old age.

A limitation of this review is that it did not involve quantifying the association between CVD risk models and cognitive function through meta-analysis. Another limitation is that we included cross-sectional studies. Furthermore, only cross-sectional studies were used to explore the correlation between CVD risk models and dementia because the latest models have not yet been used for longitudinal cohorts. We aimed to maximize the search for suitable studies on the correlation between CVD risk models and dementia, therefore, cross-sectional and longitudinal cohorts were included. It is expected that more cohorts can be conducted in the future. The aim of this analysis was not to come to any firm conclusions about risk models’ powers to predict performance on cognitive decline, but to provide an overarching viewpoint on this trend that can inform future efforts to regulate CVD and dementia and promote health.

## 5. Implications for clinical practice and policy

Growing evidence points to a tight link between heart and brain health, with CVD possibly leading to brain illnesses such as stroke, dementia, and cognitive impairment. The overall picture of the anatomical and functional connections between the heart and the brain is still unknown at this time. Early detection and prompt management of modifiable risk factors are essential for CVD or dementia, therefore, there is a need for medical professionals to focus more on health promotion.

By understanding human health from a multi-organ perspective, medical professionals can improve disease risk prediction and prevention and mitigate the adverse effects of disease in one organ on other organs at risk. Using mature CVD risk models to predict cognitive decline or dementia incidence has several implications for optimizing clinical practice in escalating care among professional staff. First, two outcomes, CVD and dementia incidence, may be acquired at a one-time assessment, simplifying the preventive services. Second, compared with CAIDE, CVD risk models have few items and few experimental indicators, which are easier to collect, and patients also have the opportunity to self-monitor and self-manage. Finally, it is critical to increase public awareness of the link between CVD and dementia and to reaffirm the significance of preventing and controlling CVD risk factors. Keeping the heart healthy can help prevent cognitive decline in non-CVD or CVD patients.

## 6. Conclusion

The current systematic review shows the rapidly spreading use of present CVD risk models to predict dementia or cognitive decline. With the rapid development of CVD risk scores, we updated Harrison’s study published in 2014 (Harrison et al., 2014). This review presents findings from a large variety of cross-sectional studies and cohorts published between 2014 and 2023, showing significant progress in this field. Our findings prove that a positive association was observed between nationally or multiethnic-based CVD risk scores and subsequent dementia or cognitive impairment. Although meta-analysis was not conducted for different models’ risk, this study supports findings that indicate that the included models may be associated with CVD, cognitive function, and dementia.

Given that these factors are easily accessible in clinical and research settings and may be used to identify the members of a population who are most at risk for future cognitive decline and dementia, future efforts should be concentrated on developing vascular factor-based dementia or cognitive decline risk models. More cohorts could clarify the link between CVD risk models and dementia or cognitive decline by unifying the outcome assessment. Additionally, constructing models applicable to low-income and middle-income countries or multiethnic populations is becoming increasingly significant.

## Data availability statement

The original contributions presented in the study are included in the article/Supplementary material, further inquiries can be directed to the corresponding author.

## Author contributions

RRJ: Data curation, Methodology, Writing – original draft. QW: Methodology, Supervision, Writing – review & editing. HYH: Data curation, Methodology, Writing – review & editing. TL: Supervision, Writing – review & editing. CFY: Supervision, Writing – review & editing. YLY: Data curation, Methodology, Writing – review & editing.

## Figures and Tables

**Table:** 

CVD	Cardiovascular Disease
BP	Blood Pressure
TC	Total Cholesterol
CAIDE	Cardiovascular Risk Factors, Aging, and Dementia
ApoE ℇ4	Apolipoprotein E ℇ4
ASCVD	Atherosclerotic Cardiovascular Disease
China-PAR	Prediction for ASCVD Risk in China
LE8	Life's Essential 8
PRISMA	Preferred Reporting Items for Systematic Reviews and Meta-analysis
OR	Odds Ratio
CI	Confidence Interval
HR	Hazard Ratio
AUC	Area Under the Receiver Operating Characteristic Curve
NOS	Newcastle-Ottawa Scale
AHRQ	Agency for Healthcare Research and Quality
PROBAST	Prediction model Risk Of Bias ASsessment Tool
NACC	National Alzheimer Coordinating Center
H/L	Hispanic/Latino
WHICAP	Washington Heights-Inwood Community Aging Project Risk Score
BMI	Body Mass Index
LS7	Life's Simple 7
AHA	American Heart Association
CANHEART	Cardiovascular Health in Ambulatory Care Research Team
TCVR	Thai Cardiovascular Risk score
KPS	Korean Coronary Heart Disease Risk Score
SCORE	Systematic COronary Risk Evaluation
ASCVD-PCE	Atherosclerotic Cardiovascular Disease Pooled Cohort Equation
GVRS	Global Vascular Risk Score
CVH	Cardiovascular Health
SBP	Systolic Blood Pressure
AD	Alzheimer's disease
MCI	Mild Cognitive Function
AD	Alzheimer's disease
VD	Vascular Disease
ICD-9, ICD-10	International Classification of Diseases Version 9 or 10
CDR	Clinical Dementia Rating
MMSE	Mini-Mental State Examination
MOCA	Montreal Cognitive Assessment
FGCRS	Framingham General Cardiovascular Risk Score
FSRP	Framingham Stroke Risk Profile
rFSRP	Revised Framingham Stroke Risk Profile
LVEF	Left Ventricular Hypertrophy
NOMAS	Northern Manhattan Study
ROC	Receiver Operator Characteristic Curve
DSC	Digit Symbol Coding
DS	Digit Span
