# Erratum: Cardiovascular disease risk models and dementia or cognitive decline: a systematic review

**DOI:** 10.3389/fnagi.2023.1355151

**Published:** 2024-01-05

**Authors:** Frontiers Production Office

**Affiliations:** Frontiers Media SA, Lausanne, Switzerland

**Keywords:** cardiovascular disease risk models, cardiovascular health, dementia, cognitive decline, health promotion

Due to a production error, Ruirui Jia and Qing Wang were not listed as sharing first authorship.

The publisher apologizes for this mistake. The original article has been updated.

